# Female genital mutilation: current awareness, believes and future intention in rural Egypt

**DOI:** 10.1186/s12978-018-0625-1

**Published:** 2018-10-17

**Authors:** Eman S. Mohammed, Amany E. Seedhom, Eman M. Mahfouz

**Affiliations:** Public Health and Preventive Medicine, Faculty of Medicine, Minia University, Minia, 1666 Egypt

**Keywords:** Female genital mutilation, Attitude, Predictors, Rural, Egypt

## Abstract

**Background:**

Female genital cutting, also termed female genital mutilation (FGM), is a damaging practice with no health benefits for girls or women, and is considered to be a violation of children’s rights.

**Methods:**

A cross-sectional, community-based study using interview administered questionnaire to explore knowledge and attitude of people living in a rural area in Minia. Systematic random sampling was used to interview 618 males and females above the age of 18 in the period from September to November 2016.

**Results:**

FGM was performed on 76.6% of females, complications occurred in 35.6% of them. Females demonstrated a higher level of knowledge than males. Nearly 56% of respondents believed that this practice should continue. Females were more supportive of the continuation of FGM than men (60.3% vs. 47.9%). The attitude that FGM is a good practice, knowledge level, women’s status and religion were significantly associated with women’s willingness to subject their daughters to FGM in the future. Attitude was the only significant predictor associated with men’s willingness to subject their daughters to FGM.

**Conclusion:**

The strong correlation between social pressure and intentions to carry out FGM means that FGM practice will continue to be embraced among future generations unless policies are put in place to eradicate this practice through empowering females by education and reasonable income.

## Plain English summary

Previous research assessed either female or male knowledge or attitude to FGM; this research explores gender perspectives and attitudes. Another important studied point is ability to explore future intention to undertake the practice to their daughters. Involving males in the battle against FGM is essential and necessitate innovative measures. Empowering females through education and reasonable income helps discouraging the practice of FGM.

## Background

Female genital cutting, also termed female genital mutilation (FGM), is a damaging practice with no health benefits for girls and women, and is considered to be a violation of children’s rights [1]. FGM is primarily widespread in certain high-risk countries including Egypt, and its rate is directly related to women’s social status and Gender Equity (GE) [2]. The age of the girls who underwent this procedure usually varies from weeks after birth to puberty [3].

FGM practice is usually driven by religious, social and cultural impetus. Many people believe that religion is the reason behind their practice of FGM. In fact, religious scholars have different views regarding FGM. Some oppose the practice while others endorse it [4]. In some societies people practice FGM because of social pressure. One study has shown to what extent people in Minia governorate might be keen to perform FGM due to their solid belief of its direct relation to modesty and virginity. On other hand, others believe that removing “unclean” parts of the girl’s body is essential to maintain femininity [5].

According to the most recent Egyptian Health Issues Survey (EHIS) (2015), 87% of all Egyptian women, in the ages from 15 to 49 have been subjected to FGM. However, the results also suggested that the rate of the practice has declined among younger women. While the percentage of women subjected to FGM in the age group 25–49 exceeds 87%, the percentage in the age group under 25 does not exceed 70% [6]. It is noteworthy that the FGM rate for women age 15–49 shown in the 2015 EHIS is relatively low (87%) when compared to the 2008 Egyptian Demographic Health Survey (EDHS) in which the rate was as high as 91% [7].

UNICEF (2013) report shows that the level of support for the continuation of FGM among men and women varies widely across countries. In Egypt, the majority of boys and men report that they want FGM to continue. By contrast, more women than men report they would like the practice to halt. This suggests that altering FGM practice should involve not only women, but also entire communities, including men, particularly in settings where men participate in the decision-making process [3]. Therefore, we aimed to investigate the sex-specific intention of parents to continue FMG practice on their daughters. It was also crucial to assess the knowledge and attitudes of women and men in rural Upper Egypt regarding FGM.

## Methods

### Study design

This community-based cross-sectional study was conducted in a randomly chosen rural area (Nazlet El-falahin), El-Minia governorate between September and November, 2016.

### Administrative design

The local municipal of Nazlet El-falahin gave its approval for the authors to obtain demographic data about the village as to the total number of residents, the total number of homes and the total number of women and men above the age of 18.

### Sample size

The sample size was calculated using EP Info version 2000 by entering the prevalence of FGM (87%), the number of women and men above the age of 18 years (6253) and the confidence level (99.99%). Accordingly, the minimum required sample size was (600); 400 females and 200 males, which represents 9.6% of the total age-eligible inhabitants.

Therefore, we used a systematic random sampling technique by numbering the houses in the village choosing the first house at random, and then every fifth house to survey an expected number of (644 participants). Data were collected from 618 participants; 418 women and 200 men who agreed to be interviewed and to participate in the study. The remaining women and men declined to participate in the study. The response rates of women and men were 96.7% and 94.3% respectively. Noteworthy, we do not have reproductive data for the 26 (14 female and 12 male) who refused to participate in the study. However, there was no significant difference between those who participated and those who refused to participate as regards the distribution of socio-demographic characteristics such as age, religion, educational level, and marital status.

A pilot study was performed on 60 persons from the same village from an area away from the sampled households to ensure their non-inclusion in the final sample and the questionnaire was further validated through a review panel process where each item was considered for appropriateness. The data for these 60 persons was not included in the study results.

The questionnaires were administered, face to face, by 10 of fourth grade medical students. The medical students had attended one week of training on FGM and social research skills before filling the questionnaires. We did not sample different households for men and women. All eligible men and women who had consented to participate were interviewed. However, in most households, the number of interviewed subjects was not more than three eligible participants. We could not match couples as husbands and wives, because of the possibility that the interviewed males could be brothers, brothers-in-law, or other relatives co-residing with the interviewed women, and vice versa. Eligible men were interviewed by male interviewers, while eligible women were interviewed by female interviewers.

### Data collection

The questionnaire was divided into four sections covering the following topics:

1. Personal characteristics and socio-demographic details including gender, age, educational level, religion and marital status.
2. The participants’ knowledge about FGM was assessed through questions about its health consequences, reasons given for performing this practice and sources of this knowledge. The total knowledge score for each participant was calculated based on the answers given to each question: “correct answer” was assigned a score of 1; “incorrect/do not know” was assigned a score of 0. Scores less than 50% (less than 5 points) were categorized as “poor knowledge level”, and scores between 50 and 100% were categorized as “good knowledge level”.
3. Participants’ attitude towards the practice of FGM was assessed using questions including: whether it should be considered a good practice, its continuation, husbands’ preference for wives subjected to FGM, discrimination against girls who did not undergo FGM, the involvement of men in the debate on FGM, whether it decreased promiscuity and whether this practice can ever be eliminated in Egypt. Participants’ attitudes were positive, negative or neutral*.*

The total attitude score for each participant was calculated based on the answers given to each question: “positive attitude” was assigned a score of 1; “neutral and negative attitude” was assigned a score of 0. Scores less than 50% (less than 4 points) were categorized as “Not in favour of FGM”, and scores between 50 and 100% were categorized as “In favour of FGM”. The eight questions that determined the attitude constituted of some negative statements, for which the scorings were reversed.

4- *The practice* was also assessed by asking if females were subjected to FGM or not, by whom, whether any complications have occurred (the interviewers mentioned the immediate and long term complications one by one) and whether they would subject their own daughters to this practice in the future.

### Statistical analysis

The Statistical Program SPSS for windows version 20 was used in data analysis. Univariate and bivariate analyses with chi square tests were conducted to detect differences in knowledge and attitude between both sexes. For multivariate analyses the dependent variable was women’s and men’s willingness to subject their daughters to FGM in the future. Risk ratios were estimated by odds ratios (OR) via both binary logistic regression and Generalized Estimating Equations (GEE) analyses. Statistical significance was set at *p* < 0.05.

## Results

The study highlighted the key characteristics that either foster or discourage the continuation of the practice. A total of 618 rural dwellers were included in the present study. Respondents’ average age was 31.8 ± 14.6 years. About 90% were Muslims, 67.6% were women and 55.3% were married (Table 1).

**Table 1:** Socio-demographic profile of studied population, rural Minia, Egypt, during September–November 2016

Socio-demographic variables	Frequency	Percentage
Sex		
Male	200	32.4
Female	418	67.6
Age		
Range (mean ± SD)	18–78 (31.8 ± 14.6)	
Educational level		
Illiterate	100	16.2
Read and write	64	10.4
1ry education	52	8.4
2nd education	164	26.5
University	238	38.5
Religion		
Muslims	558	90.3
Christian	60	9.7
Marital status		
Single	252	40.8
Married	342	55.3
Divorced	4	0.6
Widowed	20	3.2
Total	618	100

Correct answers about the reasons of FGM were significantly higher among female than among male participants. The percentage of those who did not consider FGM as a mandatory religious practice was 64.1% of females versus 49% of males, did not consider it a proper protection to virginity (41.6% versus 26%) and did not view it as hygiene for vagina (47.8% versus 32%). All these differences were statistically significant.

Evaluation of rural dwellers’ knowledge on FGM-related complications showed that 57.9% of females knew that the practice badly affected girls’ health and welfare. Correct answers that FGM causes difficulties during delivery and leaves scars were reported among 25.8% and 53.1% of females respectively compared to 10% and 38% of males respectively (*p* < 0.0001). Nearly half of female participants versus 38% of men were aware of FGM being illegal (Table 2).

**Table 2:** Knowledge of female genital mutilation among the rural dwellers, rural Minia, Egypt, during September–November 2016

Knowledge	Females no. (%) *n* = 418	Males no. (%) *n* = 200	Total no. (%) *n* = 618	X2 *P* value
Reported answers of rural dwellers about reasons of FGM				
FGM is a mandatory religious practice?				
Incorrect answer/don’t know	150 (35.9)	102 (51)	250 (40.8)	14.3
Correct answer	268 (64.1)	98 (49)	366 (59.2)	0.001
FGM is hygienic for vagina				
Incorrect answer/don’t know	218 (52.2)	146 (68)	354 (57.3)	14.9
Correct answer	200 (47.8)	64 (32)	264 (42.7)	0.001
FGM protects virginity				
Incorrect answer/don’t know	244 (58.4)	148 (74)	392 (99.4)	14.5
Correct answer	174 (41.6)	52 (26)	226 (36.6)	0.001
Reported answers of rural dwellers about consequences on health of FGM				
Bleeding				
Incorrect answer/don’t know	184 (44)	78 (39)	262 (42.4)	1.4
Correct answer	234 (56)	122 (61)	356 (57.6)	0.2
Reduction of sexual desire				
Incorrect answer/don’t know	214 (51.2)	80 (40)	294 (53.7)	8.8
Correct answer	204 (48.8)	120 (60)	324 (52.4)	0.01
Transmission of infectious diseases				
Incorrect answer/don’t know	248 (59.3)	114 (57)	362 (58.6)	0.3
Correct answer	170 (40.7)	86 (43)	256 (41.4)	0.6
Difficulty during delivery				
Incorrect answer/don’t know	310 (74.2)	180 (90)	490 (79.3)	21.6
Correct answer	108 (25.8)	20 (10)	128 (20.7)	< 0.0001
Affects health and welfare of women				
Incorrect answer/don’t know	176 (42.1)	118 (59)	294 (47.6)	15.5
Correct answer	242 (57.9)	82 (41)	324 (52.4)	< 0.0001
Scar formation				
Incorrect answer/don’t know	196 (46.9)	124 (62)	320 (51.8)	12.4
Correct answer	222 (53.1)	76 (38)	298 (48.2)	< 0.0001
There is a law against FGM				
Incorrect answer/don’t know	214 (51.2)	124 (62)	338 (54.7)	6.7
Correct answer	204 (48.8)	76 (38)	280 (45.3)	0.03
Knowledge score				
Poor knowledge level	141 (33.7)	88 (44)	229 (37.1)	6.1
Good knowledge level	277 (66.3)	112 (56)	389 (62.9)	0.01
Source of information				
Newspapers	18 (4.3)	4 (2)	22 (3.6)	17.9
TV/radio	202 (48.4)	72 (36)	274 (44.3)	0.006
Social meetings	14 (3.3)	8 (4)	22 (3.6)	
Mosque or church	10 (2.4)	2 (1)	12 (1.9)	
Friends and relatives	140 (33.5)	84 (42)	224 (36.2)	
More than one answer	34 (8.1)	30 (15)	64 (10.4)	

Of all participants studied, 37.1% had a poor level of knowledge while 62.9% had a good level of knowledge. Females demonstrated a higher level of knowledge than males (*P* = 0.01). The main source of information was television or radio which are used more often by females compared to males (*p* = 0.006).

Questions which were designed to measure the attitude of rural residents towards the practice of FGM showed that about 56% of rural residents believed that this practice should be continued. Females were more supportive of the continuation of FGM than men (60.3% vs. 47.9%). Also in this study, it was found that 47.3% Muslims versus 30% Christians (*p* = 0.01) believed that FGM is a good practice and should be continued.

Discrimination against girls who did not undergo FGM was found in 39.8% of rural residents. Discrimination was found to be higher among females than males (41.9% vs. 35.6%) but this difference was statistically not significant (Table 3).

**Table 3:** Positive and negative attitudes of FGM among male and female rural dwellers, rural Minia, Egypt, during September–November 2016

Attitude	Females no. (%)	Males no. (%)	Total no. (%)	X2 *P* value
FGM is a good practice				
Positive attitude	148 (39.5)	107 (57.8)	225 (45.5)	16.9
Negative attitude	227 (60.5)	78 (42.2)	305 (54.5)	< 0.0001
FGM should continue				
Positive attitude	245 (60.3)	92 (47.9)	337 (56.4)	8.2
Negative attitude	161 (39.7)	100 (52.1)	261 (43.6)	0.004
Uncircumcised girls should be discriminated against				
Positive attitude	170 (41.9)	69 (35.6)	239 (39.8)	2.2
Negative attitude	236 (58.1)	125 (64.4)	361 (60.2)	0.1
FGM can ever be eliminated in Egypt				
Positive attitude	185 (45.6)	111 (56.9)	296 (49.3)	6.8
Negative attitude	221 (54.4)	84 (43.1)	305 (50.7)	0.009
Men should be involved in the debate on FGM				
Positive attitude	176 (43.2)	122 (61.9)	298 (49.3)	18.5
Negative attitude	231 (56.8)	75 (38.1)	306 (50.7)	< 0.0001
Husband prefer wives subjected to FGM				
Positive attitude	191 (46.8)	140 (71.4)	331 (54.8)	32.4
Negative attitude	217 (53.2)	56 (28.6)	273 (45.2)	< 0.0001
Should FGM be criminalized				
Positive attitude	268 (66)	72 (36.7)	340 (56.5)	46.1
Negative attitude	138 (34)	124 (63.3)	262 (43.5)	< 0.0001
FGM decreases promiscuity				
Positive attitude	97 (23.8)	89 (46.1)	186 (31)	30.4
Negative attitude	310 (76.2)	104 (53.9)	414 (69)	< 0.0001

*NB* Neutral attitude was excluded from both female and male categories

Nearly 49% of the participants considered that men should be involved in the debate on FGM. This opinion was significantly more embraced among males than females (61.9% vs. 43.2%). Also, about 49% of rural residents believed that FGM could be eliminated in Egypt. Males were more supportive of this attitude (56.9% vs. 45.6%).

About 55% of rural residents believed that husbands preferred that their wives be subjected to FGM. Males tended to endorse this attitude more than females (71.4% vs. 46.8%). Nearly 57% of rural residents believed that FGM should be criminalized. This attitude was more supported by females (66% vs. 36.7%). Thirty one percent of rural residents believed that FGM reduced promiscuity. Males were more supportive of this attitude (46.1% vs. 23.8%).

Type I (clitoris or the clitoral hood is cut off) and II (clitoris and inner lips are cut off) FGM were performed on 320 (76.6%) of females. The average age of circumcision was 11.5 ± 2.3 years. The practice was performed by a doctor in only 8.7% of the cases whereas 91.3% of them were by non-medical persons. Complications occurred in 35.6% of females who were subjected to FGM, 97.2% of them have been cut by non-medical persons (Table 4).

**Table 4:** Practice of FGM among studied females, rural Minia, Egypt, during September–November 2016

Practice	Yes no. (%)	No no. (%)
Was FGM performed on you?	320 (76.6)	98 (23.4)
Was FGM performed by a doctor?	28 (8.7)	292 (91.3)
Did any complication occurs (as perceived by her)?	114 (35.6)	206 (64.4)
Age at circumcision		
Range	4–17	
(mean ± SD)	11.5 ± 2.3	
Age groups (number of women)	no. circumcised (percentage)	
< 19 (208)	136 (65.4)	
20–24 (58)	46 (79.3)	
25–29 (42)	38 (90.5)	
30–34 (26)	22 (84.6)	
35–39 (24)	22 (91.7)	
40–44 (24)	24 (100)	
45–49 (16)	14 (87.5)	
≥ 50 (20)	18 (90)	
Marital status (number of women)	no. circumcised (percentage)	
Never married (230)	148 (64.3)	
Ever married (188)	172 (91.5)	
Total	418	100

Table 5 provided the adjusted OR and 95% CI that quantified the association between the combined effect of independent variables and the outcome variable (women’s willingness to subject their daughters to FGM in the future). These estimates were obtained using the logistic regression analysis. Attitude that FGM is a good practice, knowledge level, women’s status and religion were significantly associated with women’s willingness to subject their daughters to FGM in the future. The results suggest that while education is not significantly associated with women’s willingness to subject their daughters to FGM, attitude that FGM is a good practice represented the major cause.

**Table 5:** Logistic regression of factors independently associated with women’s willingness to subject their daughters to FGM in the future, rural Minia, Egypt, during September–November 2016

Risk factors	OR (95% CI)	*p*
Women status		
No FGM	1.00 (Reference)	0.004^a^
Complicated FGM	0.07 (0.02–0.34)	
Not complicated FGM	1.13 (1.02–1.97)	
Attitude that FGM is a good practice		
No	1.00 (Reference)	0.0001^a^
Yes	2.35 (1.73–8.95)	
Knowledge level about FGM		
Good	1.00 (Reference)	0.001^a^
Poor	1.28 (1.14–1.59)	
Religion		
Christian	1.00 (Reference)	0.03^a^
Muslim	1.02 (0.93–1.25)	
Educational level		
University level	1.00 (Reference)	0.2
Secondary education	0.17 (0.04–0.81)	
Basic education	0.89 (0.1–4. 92)	
Illiterate	1.28 (0.2–3.54)	
Marital status		
Never married	1.00 (Reference)	0.07
Ever married	0.82 (0.4–1.23)	
Age group		
< 25 year	1.00 (Reference)	0.84
> 25 year	1.83 (0.53–4.21)	

Cox & Snell R Square = 0.56

NB dependent variable is women’s willingness to subject their daughters to FGM in the future, OR odds ratio, CI confidence interval

^a^Statistically significant

Table 6 showed logistic regression analysis of the independent variables and the outcome variable (men’s willingness to subject their daughters to FGM in the future). Attitude that FGM is a good practice was the only significant factor associated with men’s willingness to subject their daughters to FGM in the future.

**Table 6:** Logistic regression of factors independently associated with men’s willingness to subject their daughters to FGM in the future, rural Minia, Egypt, during September–November 2016

Risk factors	OR (95% CI)	*p*
Attitude that FGM is a good practice		
No	1.00 (Reference)	0.0001^a^
Yes	1.63 (1.07–2.89)	
Spouse’ status		
No FGM	1.00 (Reference)	0.32
Complicated FGM	1.75 (0.15–20.3)	
Not complicated FGM	8.58 (0.38–196.2)	
Knowledge level about FGM		
Good	1.00 (Reference)	0.82
Poor	1.84 (0.19–3.71)	
Religion		
Christian	1.00 (Reference)	0.87
Muslim	1.25 (0.09–16.9)	
Educational level		
University level	1.00 (Reference)	0.6
Secondary education	0.14 (0.01–1.78)	
Basic education	1.31 (0.17–12.8)	
Illiterate	1.94 (0.11–7.74)	
Marital status		
Never married	1.00 (Reference)	0.9
Ever married	0.78 (0.02–41.3)	
Age group		
< 25 year	1.00 (Reference)	0.2
> 25 year	1.1 (0.003–3.67)	

Cox & Snell R Square = 0.50

NB dependent variable is men’s willingness to subject their daughters to FGM in the future, OR odds ratio, CI confidence interval

^a^Statistically significant

## Discussion

Researcher’s attention has increasingly been directed towards FGM over the last decades due to its effect on women’s health representing a severe violation of a basic human right: bodily integrity [8]. Previous studies have shown that FGM occurred among females in all social strata, uneducated and highly educated, urban and rural residents [9]. Studies on FGM among the general population in Egypt have reported that 87% of Egyptian women have been subjected to FGM [10, 11].

Evaluation of rural residents’ knowledge on FGM-related complications showed that 57.9% of females knew that the practice badly affected girls’ health and welfare. Similar finding was reported by Moges et al. (2014) in a study conducted to assess knowledge of women towards FGM in Ethiopia [12].

About 55% of rural residents had knowledge that FGM was against the law, which was similar to that of Emam et al. who noticed that despite women’s awareness that FGM was illegal, they would still practice it. This showed that the law alone was not sufficient to eradicate this habit, and it seemed that laws forbidding FGM led to changes in the patterns of the practice [13].

Eventually, of all participants studied, 37.1% had a poor level of knowledge and 62.9% had a good level of knowledge. Females demonstrated a higher level of knowledge more than males (*P* = 0.01). This finding was in agreement with Abdel Magied and Makki, 2004 who found that female respondents were far more knowledgeable about the negative consequences of FGM [14].

Our results showed that females were more supportive of the continuation of FGM than men (60.3% vs 47.9%) because females are greatly affected by the culture that supports inequality between men and women. In contrast to this, the study done in Somalia reveals that male respondent (96%) supported the continuation of FGM. This difference may be explained by the fact that the achieved progress towards the abandonment of the practice in Somalia has been known to be very limited due to the unprecedented prevalence of the practice in Somalia [15].

In the current study, 54.8% of rural residents believed that husbands preferred that their wives being subjected to FGM. Males were more supportive of this attitude (71.4% vs. 46.8%). This was not in coherence with Abathun et al. who found that more than half of the studied young boys preferred to marry girls not subjected to FGM. This implies that young boys had unrestrained freedom to obtain knowledge about the harmful effect of FGM on sexuality [16].

The overall prevalence of FGM among female participants in this study is considered to be low when compared with the 2015 DHS data [6]. Yet, as data disaggregated by age, the higher prevalence of FGM is evident for the older age group of females (> 25 years) rather than younger age group. In the current study, more than half of the studied females aged below 25 years; meanwhile, nearly 90% of females in the older age group were found to be subjected to FGM, which is more or less approaching what was reported by the national EHIS, 2015.

By comparing the percentages of those who had reported living with FGM across different age categories in the current study with the respective percentages of the EHIS, 2015, FGM prevalence in our study was slightly lower in the age categories (15–19 years 65.4% vs. 69.6%) and (20–24 years 79.3% vs. 81.6%) and (35–39 years 91.7% vs. 95.4%) and (45–49 years 87.5% vs. 97.1%); but was slightly higher in the age categories (25–29 years 90.5% vs. 89.2%) and (40–44 years 100% vs. 94.9%). Likewise the EHIS, 2015, we found that the highest prevalence of FGM was in the age groups (35–39) and (25–29) years; both came the second and third top percentages in both studies respectively. The percentages of FGM among the never- married females in our study was lower than that among ever-married females (64.3% vs. 91.5%), which are found to be slightly lower than the findings of EHIS, 2015 (67.6% vs. 91.5%) [6].

The practice was performed by a doctor in only 8.7% of the cases while 91.3% of them were by non-medical persons. This result was inconsistent with the finding of Rasheed et al. which was carried out also in Upper Egypt and aimed to estimate the influence of the 2007 criminalization law on the prevalence and yearly incidence of FGM, and it was found that in their vast majority, the procedures were performed by general practitioners. In total, 88.2, 34.3 and 14.9% of nurses, young physicians, and senior physicians, respectively, approved the practice. This difference may be explained by the fact that the study of Rasheed et al. was performed from September 2008 to September 2010, shortly after the criminalization law was enacted in Egypt, so physicians were unaware of the consequences of this criminal action [17].

The logistic regression analysis showed that knowledge, attitude, religion and women’s status are significant predictors of future FGM practice among the daughters of females, with attitude that FGM is a good practice representing the major cause. These findings were in agreement with Pashaei et al. who examined the determinants of women’s intentions to subject their daughters to FGM, and it was found that attitude was the strongest predictor of perpetuation of FGM [18]. Therefore, intervention programs to decrease FGM might focus primarily on converting women’s positive attitude toward FGM into negative attitude, and on alleviating the perceived social pressure to mutilate one’s daughter [19].

Men’s contribution in the prevention of this harmful practice is indeed needed. The prospects of eliminating FGM will most likely to come to an end if due attention is given to male attitudes [20]. In a rural Upper Egyptian society, like our study focus, where the majority of men lead, and decide for women who to interact with and who to socialize with, it is highly advised that men be included in all efforts to prevent/eliminate FGM adequately [21]. However, researches involving men’s future intentions and their influence on the perpetuation of the FGM are limited.

This study found that the attitude was the only significant predictor for men’s willingness to subject their daughters to FGM; so we recommend conducting health education sessions to the rural educated men, and consequently they will most likely help to change positive attitudes of none and low educated men in their families towards continuation of FGM. Television and other media have an important role in changing the attitude of rural men and so contribute to discontinuation of FGM.

This study revealed that the participants were trapped in customary traditions, and found out controversy and divergence between the participants’ perception of the harmful effects of FGM and their inclination towards maintaining FGM, giving an impetus to advance strong measures against the harmful practice. A proper measure in this regard is the Transtheoretical Model which facilitates the process of intentional behavior change in different community settings.

People can move through a series of stages during behavior modification to reduce resistance, facilitate progress, and prevent relapse of the FGM behavior [22]. The stages include: precontemplation (not ready), contemplation (getting ready), preparation, action and maintenance. As population move from *preparation* to *action*, the gap between sense of self-efficacy and temptation gets closer, and behavior change is achieved. So, to redress these harmful behaviors, people need to become aware of the possibility of change, have a favorable opinion towards change, choose to adopt or reject the change, put change into practice, and reinforce or reject change when faced with conflict or difficulty [22].

### Limitations of the study

Some women and men refused to participate in the study. The response rates were 96.7% and 94.3% for women and men respectively. Refusal of participation could be attributed to their sense of shame, or because FGM is an illegal practice. The possibility to recall bias, or reporting bias was another limitation. Despite the fact that accuracy of self-reported data can be compromised by real ignorance about status or purposeful misrepresention of their status, yet, the validity of the results was not affected by this type of bias as it did not exceed 8% in previous studies in Egypt between the reported FGM and actual status. Because circumcision is a sensitive issue, our data collectors were skillful and used appropriate procedures adjusted for participant’s response.

Another point worth mentioning is the fact that often more than one participant was recruited from the same household and therefore there is a possibility for violation of the independent observation assumption of logistic regression analysis. We dealt with this issue in several ways, first we checked if the distribution of number of interviewed participants per a household among each group of outcome (willing to subject their daughter to FGM versus not willing to do so, and we found no significant difference in this distribution. Second, we addressed this clustering of the measurements through the empirical variance estimation method (the robust standard error method) by running GEE analysis with robust covariance matrix in SPSS. This GEE analysis yielded more or less similar parameter estimates (OR) as those obtained by the binary logistic regression.

## Conclusions and recommendations

Women’s knowledge, attitude, religion and status are proved to be significant predictors of the women’s future intentions to carry out FGM practice on daughters, thereby, concrete efforts need to be exerted in these specific areas, particularly through securing and realizing quality education to the fullest potential, offering explanatory role by religious leaders that FGM is neither mandated nor recommended by either Islam or Christianity, and approaching females by health education messages in schools, MCH and in any other opportunity of contact.

Female genital mutilation needs to be reframed within the broader issue of gender inequity and inequality which cuts across geography, socioeconomic, religion and education. Promoting gender justice and equality through taking actual steps towards empowering women and improving their position and status by ensuring quality education will enhance decision making capacity and will pave the way towards elimination of the FGM practice.

The findings of the study highlighted the welcoming attitude of men towards the continuity of FGM practice. So sincere scientific efforts of changing men’s attitudes are greatly needed through involving them seriously and adequately in all efforts of prevention and elimination of FGM.

It is essential to strengthen and scale up the momentum for change brought about by the recent issuing of a law that banned and criminalized the FGM act, especially in rural areas in Upper Egypt, in order to turn the potentials into actual changes in attitudes and behaviors.

### Implications of the study

It’s important to train health care providers on how to address the complications of FGM and to make sure that the concerned health services are able to deal with these cases.

## Abbreviations

EDHS: Egyptian Demographic Health Survey.
EHIS: Egyptian Health Issues Survey
FGM: Female genital mutilation
GE: Gender Equity
